# Can co-designing interventions with affected communities help prevent violence against women? Findings from a process evaluation of the *E le Saua le Alofa* (Love Shouldn’t Hurt) pilot in Samoa

**DOI:** 10.1093/heapol/czag009

**Published:** 2026-02-04

**Authors:** Jenevieve Mannell, Hattie Lowe, Helen Tanielu, Ene Isaako Hosea, Pepe Tevaga, Louisa Apelu, Fa’afetai Alisi Fesili, Andrew Copas

**Affiliations:** UCL Institute for Global Health, 30 Guilford Street, London, WC1N 1EH, United Kingdom; Centre for Samoa Studies, National University of Samoa, To'omatagi, Samoa; UCL Institute for Global Health, 30 Guilford Street, London, WC1N 1EH, United Kingdom; Department of Social Science, National University of Samoa, To'omatagi, Samoa; Samoa Victim Support Group, Apia, Samoa; Samoa Victim Support Group, Apia, Samoa; Independent consultant, Apia, Samoa; Samoa Victim Support Group, Apia, Samoa; UCL Institute for Global Health, 30 Guilford Street, London, WC1N 1EH, United Kingdom

**Keywords:** co-production, violence against women prevention, interventions, mixed methods, pilot evaluation, Samoa

## Abstract

There has been increasing interest in co-designing interventions with end users to prevent violence against women (VAW). Co-design is theorized as an ethical approach to research able to engage some of the most marginalized groups in VAW prevention. However, there is little evidence of whether co-designing interventions can reduce violence against women, or a theoretical consideration of *how* it might do so. This paper contributes to current discussions about co-design by examining the results of the *E le Saua le Alofa* (Love Shouldn't Hurt)—a pilot intervention that engaged Samoan communities in co-designing violence prevention activities. A mixed-methods evaluation of the pilot has shown promising results, and in this paper, we consider how the co-design process may have contributed to these results. The evaluation of the co-design process assessed four theorized mechanisms: (1) increased ownership of the problem of violence; (2) improved health behaviours and social norms; (3) relevance of actions taken to address VAW; (4) addressing power structures arising from coloniality. Our results show that a change in violence outcomes occurred through the pilot's ability to revisit previous conversations about violence in Samoa, prompting new activities by local leaders, and tightening village rules on violence. Yet, the activities implemented by local leaders were largely unpredictable and sometimes conflicted with global evidence. We argue that such actions should not be construed by policymakers as the “unpredictable outcomes” of an intervention, but rather understood within a broader framework of diversified knowledge systems. The need for balance in co-designing VAW interventions with communities affected by violence highlights a key challenge of decolonizing VAW practice within a co-production framework.

Key messages

- This study contributes theoretically to the co-design literature by evaluating the co-design process of a pilot intervention to reduce violence against women in Samoa.
- We assess the co-design process using mixed methods data defined by four theorized mechanisms from the co-design literature.
- The success of the intervention (although not definitive) was achieved through instigating new conversations and actions by local leaders in villages about violence, highlighting the potential of south-to-south knowledge exchange as part of a co-design process.

## Introduction

The negative impacts of violence against women (VAW) on the physical and mental health of women are well established (Garcia-Moreno et al. 2006, Oram et al. 2022). Globally, one quarter of women will experience violence in their lifetime, and in some regions of the world, this figure is closer to one in two (Mannell et al. 2022 , Sardinha et al. 2022 ). There is also growing evidence for how to prevent VAW, highlighting the potential of achieving VAW reductions within programme timeframes of 4–5 years (Heise 2011, Jewkes 2014). Community-focused interventions such as *Stepping Stones* in South Africa, *SASA!* in Uganda, and *MAISHA* in Tanzania have been instrumental in expanding this baseline to low- and middle-income countries (LMICs) (Abramsky et al. 2016, Kapiga et al. 2019, Gibbs et al. 2020).

Despite these successes, VAW prevention has been challenging for some of the world's most marginalized communities, including young people living in urban informal settlements, undocumented migrants, and Indigenous communities (Thomas et al. 2022). To identify potential solutions for these groups, some VAW scholars have shifted towards co-designing intervention components *with* communities rather than designing interventions *for* communities. This has included engagements with Indigenous communities to co-design theories of change (Mannell et al. 2023a); training peer researchers to facilitate intervention activities in informal settlements (Mannell et al. 2023b); embedding cycles of reflection and feedback with participants as part of interventions (Daruwalla et al. 2019 ); defining and promoting healthier masculinities (Donnar et al. 2023); and adapting guidelines for healthcare providers to respond to VAW (Gadappa et al. 2022). This draws on growing evidence that co-designing interventions with end users is not only more ethical, but can lead to improved health outcomes (Brett et al. 2014, Tembo et al. 2021).

However, *if* and *how* co-designed interventions improve VAW outcomes remains unclear. Co-design is increasingly used, but rarely evaluated as either a mechanism or outcome of VAW prevention interventions (Domecq et al. 2014, Slattery et al. 2020, Mannell et al. 2026b). Our aim is to contribute to the deeper theorization of co-design for VAW prevention policy with the process evaluation of *E le Saua le Alofa* (‘Love Shouldn't Hurt’): an intervention that engaged Indigenous Samoan communities in co-designing their own VAW prevention activities.

## Theoretical engagements in co-designing interventions

Much of the debate in co-design focuses on defining what it is and is not, and developing key frameworks for its improvement (Filipe et al. 2017, Greenhalgh et al. 2019, Rose and Kalathil 2019). In contrast, we ask what it *does*. What are the expected outcomes of the co-design process? And can these outcomes be used to evaluate the ‘success’ of co-designed VAW interventions?

In public health policy, co-design is often seen as a means of improving the relevance of an intervention to a group of users (Bleijenberg et al. 2018, Staniszewska et al. 2018). However, scholars also recognize it as a process in and of itself that has the potential to change social norms and existing health practices before an intervention has even started. For example, in co-designing a VAW prevention intervention for young people living in informal settlements in South Africa, the Siyaphambili Youth study highlights how the co-design process changed not only participant's understandings of gender norms and the forms of masculinity that drive violence but also the researchers' ideas of what can and should be measured (Mannell et al. 2023b). In this vein, co-design is seen as a ‘social space’ able to create new communities, interactions, and practices that go beyond specified outcomes (Filipe et al. 2017).

A growing body of literature discusses the potential for co-design to be used as part of the process of ‘decolonizing’ VAW research practice (Breton 2023, Lokot et al. 2024). This recognizes that research practices that do not offer concrete benefits to participating communities can further perpetuate cycles of research malpractice (Smith 1999) in ways that denigrate Indigenous knowledge and ways of knowing (Suaalii-Sauni and Fulu-Aiolupotea 2014). Co-design thereby offers the potential for a more radical approach that recognizes the plurality of knowledge systems, paving the way for Indigenous communities to set their own research agenda and priorities (Mannell et al. 2021). However, for co-design to deliver on the promise of decoloniality, it needs to fundamentally address the power inequalities created and reproduced by colonial matrices of power (Burgess 2023).

## VAW prevention in Samoa

Despite Samoa's cultural strengths, including the enduring Samoan culture represented by the *matai* (chief) system, *aiga* (extended family), and *fa’a Samoa* (the Samoan way), the country faces significant challenges regarding VAW. Approximately 31.8% of women report having experienced physical, sexual, or emotional violence within the past year (Samoa Bureau of Statistics 2020). These statistics reflect the global crisis of VAW, which is particularly high in postcolonial climate-affected countries (Brown et al. 2023). However, the high prevalence of VAW can obscure existing efforts and cultural mechanisms that address and prevent violence (Lowe et al. 2023). Samoan communities have long had mechanisms for dealing with interpersonal conflict, and village councils, composed of *matai*, often deliberate on cases of domestic violence and seek resolutions that are rooted in cultural values of respect, service, and Christian values.

## The EVE project

In this paper, we discuss the process evaluation of a 1-year pilot of *E le Saua le Alofa*, developed as part of the EVE Project (Mannell et al. 2021). The intervention was co-designed with 30 community-based researchers (CBRs) from 10 Samoan villages (1 man, 1 woman, 1 elder; *n* = 30), who were first engaged in a series of research workshops led by the Samoa Victim Support Group (SVSG) and National University of Samoa (NUS) over a three year period (2020–2023). With methodological guidance provided by University College London (UCL), CBRs conducted peer interviews with members of their village about community responses to VAW, organized a village survey to assess risk and protective factors for VAW, and developed a theory of change about how VAW could be prevented in Samoa.

Findings from the initial 3 years of the project were then used to develop the pilot intervention. Drawing on the theory of change created by CBRs, the risk/protective factors identified from survey data in Samoan villages, and a scoping of evaluated intervention manuals available from other similar neo-colonial contexts, SVSG and UCL developed a preliminary manual of VAW prevention activities. This preliminary manual was then tested and iteratively refined with CBRs following principles of participatory action research (PAR): an iterative cycle of experiential learning and action where new knowledge is produced through learning from the previous stage (Brydon-Miller 1997, Cornish et al. 2023). We completed three iterative cycles that involved CBRs in: (i) testing potential activities from evidence-based interventions used in other neo-colonial contexts, (ii) modifying intervention activities for the Samoan context, (iii) implementing activities in participating communities, and (iv) reflecting on the process as a group.

We refer to ‘intervention activities’ in this paper as the set of manualized interventions that have previously been rolled out in at least one other country and had been evaluated with positive results published in peer-reviewed journals. In contrast, the ‘co-design pilot’ refers to the iterative process of co-designing a Samoan approach to VAW prevention in partnership with CBRs and their communities, including actions taken by community members during the pilot. The use of evidence-based interventions provided a means of generating dialogue about the effectiveness of community actions in the Samoan context but avoided imposing ideas about how to prevent VAW. Communities were free to develop VAW prevention actions that they saw as inherently valuable for their community without restriction.

**Figure 1:**
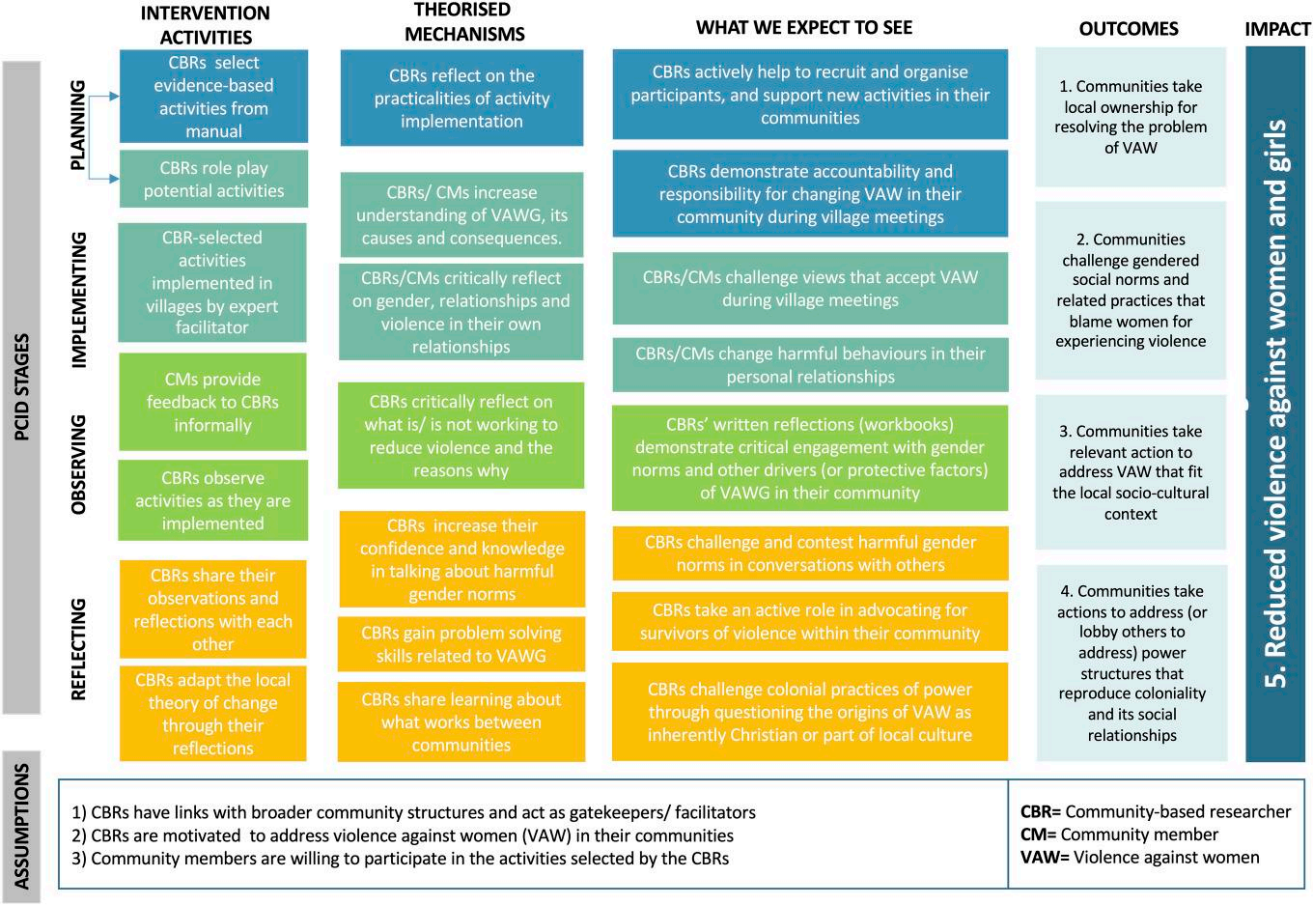
Programme theory.

The results of an acceptability and feasibility study of the pilot are reported elsewhere, including early findings of its potential impact (Mannell et al., 2026a). As a complement to the acceptability and feasibility study, this paper aims to use the same dataset to explore the programme's theoretical goals, and how it may have achieved reductions in VAW. These theoretical goals were initially developed by SVSG and UCL based on the co-design literature to explicitly clarify assumptions about *how* co-design could reduce VAW (Fig. 1).

The programme theory links pilot activities to mechanisms and co-design outcomes (what we expect to see), to explicitly measure the co-design process independent of its health evaluation (Mannell et al. 2026b). For example, having CBRs and their communities select evidence-based activities encourages personal reflection on the cultural relevance of the activities for their community and the practicalities of implementation. This was theorized to translate into greater ownership, accountability, and responsibility by CBRs and members of the community for VAW reductions, and consequently fewer cases of VAW. From the links between pilot activities, mechanisms, and early outcomes, we then produced a list of four overarching outcomes of the co-design process, which are assessed in this paper.

## Materials and methods

### Individual selection and recruitment

Our evaluation of the pilot took place between March 2023 and March 2024. We used stratified purposive sampling for this study, consistent with implementation studies of this kind (Palinkas et al. 2015). SVSG first selected 10 villages in 2020, based on diversity across the number of violence cases reported to SVSG since 2005, traditional village structure, and urban/rural location. Two CBRs from each village were recruited from the organization's network of over 1000 community representatives. SVSG has pre-existing relationships with village councils and local chiefs, which facilitated the agreement of local gatekeepers. CBRs then selected 30 members from their village to participate in the pilot. Any member of the village aged 18 or above was eligible to participate, however, CBRs were asked to invite a total of 30 individuals per village with an equal number of men and women, representatives from elders and youth, and those holding different titles (including chiefly titles and untitled men and women). The study offered a small sum to participate ($20 tala per session; ∼6GBP) and community members signed a consent form on day one.

### Data collection

#### Qualitative data

Fourteen semi-structured interviews with CBRs were conducted at the end of the pilot (February 2024) by an independent researcher, hired by SVSG and known to the CBRs. The researcher used a topic guide to ask questions about changes in their village/personal lives since the beginning of the pilot, and logistics, including why people may not have attended workshops. The SVSG team also conducted informal interviews with 151 members of pilot communities at the end of the pilot (64 men, 85 women). These involved asking individuals to share their views of the intervention and any significant changes in their village they had observed that could be attributed to the intervention. The team took detailed notes of these interactions, capturing verbatim quotes when possible, and recording these in an Excel spreadsheet. Eighteen of the 30 CBRs also completed a workbook over the course of the pilot, which was designed to guide the process of selecting, planning, and reflecting on activities for their communities, with worksheets and practical tools. All interviews and workbooks were completed in Samoan and later transcribed and translated into English.

#### Quantitative data

A survey questionnaire was given to all participants who attended the first day of the pilot before activities began, with a follow-up survey delivered to the same individuals 8–12 weeks after pilot workshops ended (44–48 weeks after the start of the pilot period). The questionnaire assessed women and men’s experience/perpetration of physical, sexual, emotional, and economic violence in the last 6 months, bystander interventions, community trust of CBRs, views on gender roles and violence, and demographics (age, education, village status, and religion). The survey was self-administered on tablets in Samoan with team members standing nearby to assist participants with any technical difficulties or to answer questions.

Women’s experiences of intimate partner violence (IPV) were assessed using questions from the Demographic and Health Multiple-Indicator Cluster Survey (DHS-MICS) to provide comparability with nationally representative data. Women were asked questions about whether they had experienced seven acts of physical IPV: being pushed, shaken, or having something thrown at them; slapped; arm twisted or hair pulled; punched; kicked, dragged, or beaten up; choked or burned; threatened or attacked with a knife. They were also asked about three acts of sexual IPV: physically forced sex; physically forced sexual acts; forced with threats to perform sexual acts, and three acts of emotional IPV: humiliated; threatened with harm; insulted or made to feel bad. To assess economic violence, we reduced the SEA-12 scale developed by Postmus et al (2016) to five items, including: being prohibited from getting a job or earning money; taking earnings against their will; refused money for household expenses; being excluded from financial decisions; debt built up under their name. Response options for all questions were often, sometimes, or not in the past 6 months. Responses to the physical, sexual, emotional, or economic IPV questions were categorized into a binary variable as either having experienced or not one of these forms of violence in the past 6 months, which served as outcome/dependent variables in the logistic regression analysis performed.

Questions about bystander interventions and the effectiveness of CBRs drew on a list of 10 statements developed from our previous work (Lowe et al. 2022) and knowledge of the Samoan context. The trust of CBRs by community members was assessed with five questions related to whether they trusted CBRs to make good decisions about VAW. Agreement with each statement was captured by response categories including strongly agree/agree/neither agree nor disagree/disagree/strongly disagree. The GEM scale was used to ask questions about views related to gender roles and violence, composed of: (i) statements accepting of violent behaviours and (ii) statements about gender norms. Agreement with each statement was captured by response categories, including strongly agree/agree/disagree/strongly disagree. Responses were transformed into a binary variable for analysis (agree/disagree).

### Data analysis

We analysed the qualitative data through a collaborative and iterative process that engaged all co-authors in framework analysis (Gale et al. 2013). Concretely, this involved an initial coding of our data deductively to assess whether the programme achieved its four theorized outcomes (Table 1), followed by a series of in-person meetings where we discussed whether we had achieved each outcome in whole/in part/or not at all. As part of these discussions, we reflected critically on our own positionality in the research process and how this may affect our analysis of the data (Sultana 2007).

**Table 1:** Data analysed for each outcome theorized.

Theorized outcome	Relevant data collected	Analysis performed
1. Communities take local ownership for resolving the problem of VAW	Interviews with CBRs CBR participation data Pre/post measure of trust of CBRs by community members	Deductive coding/collaborative analysis Descriptive analysis
2. Communities challenge gendered social norms and related practices that blame women for experiencing violence	Interviews with community members Interviews with CBRs Interviews with faciliators Pre/post measures of gendered social norms Pre/post measures of bystander interventions	Deductive coding/collaborative analysis Logistic analysis of GEM scale and bystander interventions (reported as odds ratios or % change, as appropriate)
3. Communities take local actions to address VAW that fit the local socio-cultural context	Interviews with community members Interviews with CBRs Pilot participation data	Deductive coding/collaborative analysis Descriptive analysis
4. Community take action to address (or lobby others to address) power structures that reproduce coloniality and its social relationships	Interviews with community members Interviews with CBRs	Inductive/deductive coding/collaborative analysis

Quantitative survey data were analysed in STATA 18 to further develop results around our theorized mechanisms and potential VAW reductions. We compared responses between pre and postsurveys using Chi-squared tests. While many participants at endline also completed a baseline survey, we were unable to reliably match surveys to conduct matched-pairs testing. We ran logistic regressions using the *logistic* command in STATA to measure change in outcomes between pre and postsurveys, adjusting for variables supported by available evidence as potential confounders (i.e. age, education, village), noting that not all individuals surveyed preintervention completed a survey again at follow-up. The difference between pre and postsurveys was reported as odds ratios with 95% confidence intervals and presented at co-author meetings for interpretation within the Samoan context.

## Results

Our results are presented according to the four outcomes theorized in our programme theory, merging quantitative and qualitative data with ethnographic observations collected by the first author. The capacity of the pilot to achieve theorized outcomes differed between outcomes and villages, and we have attempted to build a detailed conceptual picture of how well the co-design process worked or did not work according to the data, alongside a careful reading of the context. Demographic data for the quantitative survey are provided in Table 2.

**Table 2:** Demographic data.

	*N* (*missing*)	Women %	Men %
Total participants	289	55.7	44.3
Age	***(2)***		
18–24 (youth)	37	5.0	22.7
25–39	83	35.2	21.1
40–49	70	27.7	20.3
50–59	59	20.1	21.1
60+	38	12.0	14.8
Education	***(4)***		
Never attended	1	0.0	0.8
Early childhood education	3	1.3	0.8
Primary	19	2.5	11.9
Secondary	218	82.4	69.0
Higher	44	13.8	17.5
Status	***(53)***		
*Matai* (Village chief)	122	58.5	43.4
*Nofotane/Faiava* (women/man married into a different village community)	48	27.7	11.3
Religious leader (e.g. minister, deacon)	4	1.5	1.9
Partner of religious leader	1	0.8	0.0
Untitled	61	11.5	43.4

A total of 289 individuals completed the preintervention survey (55.7% identified as women, while 44.3% identified as men). Proportionally, the intervention had a large percentage of *matai* (local leaders) (43.4% of men; 58.5% of women), but only a small number of religious leaders (1.9% of men; 1.5% of women).

### Increased ownership of the problem of violence by CBRs

The level of CBR involvement, as an indicator of ownership, differed between villages. Sixteen of the 30 CBRs were categorized by SVSG as very active (participated in nearly all activities), 10 as active, and 4 as not very active due to frequent absences. One of the facilitators shared the observation that it was during the workshops where CBRs tested intervention activities that they also shared local village experiences of violence and how this was handled in their community. The facilitator perceived this sharing across villages as instrumental in building local ownership over intervention activities:

[The CBRs] had to dig deep and engaged meaningfully with their counterparts to really bring out the best fit for their own respective communities. It was a time where lessons were shared and talanoa sessions were vibrant [with CBRs] bringing vital narratives from their own villages and districts that supported the lean towards a certain activity. It built more awareness and brought a wealth of information that other CBRs would draw on to support their own intervention activities for their villages. (Female EVE Facilitator)

In interviews, CBRs confirmed that they would intervene in cases of VAW with strong verbal support for women experiencing violence:

If someone were to express the belief that a woman deserved to be abused, I would firmly disagree with that perspective. Abuse is never justified or acceptable under any circumstances. Instead, I would try to understand their perspective, educate them about the serious consequences of abuse, and emphasise the importance of empathy and support for victims. Additionally, I would offer resources and assistance to the individual experiencing violence. (Female CBR, village 07)

Part of the role of CBRs was to support women experiencing violence, and they had been highly trained over the four years of the EVE Project to do this. This was also demonstrated in the high level of trust CBRs had from community members to respond appropriately to VAW-related cases, which was over 90% from the beginning of the pilot. While there was no significant change in respondents’ perceptions of CBRs, there was also little room for improvement. However, while the majority of CBRs emphasized the need to support women experiencing violence, not all did, even after substantial involvement in the project. During a final interview with the project team, a male CBR discussed talking to the woman experiencing the abuse to see if she could change her behaviours, which were seen as a cause of the violence:

Interviewer: Say, someone says this to you, ‘woman deserved to be abused, it was right for her to be abused,’ what would you say to the person that said that?

Participant: I would call for the mother that was abused to come and have a chat, and we will resolve whatever is going on, reasons why that other person was yelling, and see why that person is doing these things, tell her to avoid those things, and to love her family and her children truly. (male CBR, village 09)

This individual had been involved in the EVE Project since 2022 and was an elder in his village. This demonstrates the challenge of changing people’s assumptions about the drivers of violence in highly patriarchal contexts where such views are normalized, even with substantial training.

#### Improved health behaviours and social norms

Survey results showed that the acceptance of norms of violence had potentially reduced postintervention across four of the five statements, and more so among women than men. The largest reduction was for the statement ‘*I think there are times when a woman deserves to be beaten*,’ which reduced from 23.6% to 17.1% among women (*P* = .167), and from 17.9% to 8.9% among men (*P* = .063) (Table 3).

**Table 3:** Participant views on violence at baseline and endline.

Participant agrees with the statement…	Baseline *n* (%)	Endline *n* (%)	*P*-value	Baseline *n* (%)	Endline *n* (%)	*P*-value
	Women (*N* = 301)			Men (*N* = 217)		
I think that there are times when a woman deserves to be beaten.	38 (23.6%)	24 (17.1%)	.167	23 (17.9%)	8 (8.9%)	.063
I think that a woman should tolerate violence in order to keep her family together.	22 (13.7%)	14 (10.0%)	.328	17 (13.9%)	7 (7.9%)	.211
I think that it is alright for a man to beat his wife if she is unfaithful.	25 (15.5%)	22 (15.7%)	.965	8 (6.3%)	7 (7.9%)	.645
I think that if someone insults a man, he should defend his reputation, with force if he has to.	43 (26.7%)	36 (25.7%)	.845	45 (35.2%)	28 (31.5%)	.571
I think that violence is a private matter that shouldn't be discussed outside of the couple.	39 (24.2%)	34 (24.3%)	.990	35 (27.3%)	21 (23.6%)	.535

This was confirmed by one of the facilitators of the pilot, who described how they achieved this change in social norms:

We would probe questions and then bring bible phrases to support our views as Samoans almost always believe in what the Bible says. (Female EVE facilitator)

Statistically significant reductions postintervention in women's agreement with two statements on the GEM scale were observed, namely: ‘*I think that a woman’s most important role is to take care of her home and cook for her family*’ (8.1% reduction, *P* = 0.049) and ‘*I think that changing diapers, giving the kids a bath, and feeding the kids are the mother’s responsibility*’ (8.1% reduction, *P* = 0.049). In contrast, men's agreements with the GEM scale statements did not show significant changes, although this could also be related to the small number of responses from men to this question (Table 4). Interestingly, women's gender views appear to have changed during the pilot, but not men's. In contrast, men's behaviours related to the perpetration of physical violence (mentioned previously) may have changed.

**Table 4:** Participant views on gender norms at baseline and endline.

Participant agrees with the statement…	Baseline *n* (%)	Endline *n* (%)	*P*-value	Baseline *n* (%)	Endline *n* (%)	*P*-value
	Women (*N* = 301)			Men (*N* = 217)		
I think that a woman's most important role is to take care of her home and cook for her family.	**143** (**88.8%)**	**113** (**80.7%)**	.**049**	72 (56.3%)	51 (57.3%)	.878
I think that changing diapers, giving the kids a bath, and feeding the kids are the mother's responsibility.	**143** (**88.8%)**	**113** (**80.7%)**	.**049**	90 (70.3%)	56 (62.9%)	.254
I think a man should have the final word about decisions in his home.	141 (87.6%)	115 (82.1%)	.187	99 (77.3%)	73 (82.0%)	.403
I believe it is God's will that a man is the head of the family.	154 (95.7%)	129 (92.1%)	.200	111 (86.7%)	78 (87.6%)	.842

Bolded values are statistically significant at the 5% level.

Some increases in bystander interventions were also seen, with respondents reporting an increase in eight of the ten questions. While the change was not statistically significant for any question, the mean percentage increased on all but two measures (i.e. speaking out against violence in the village; participating in a village or church activity on reducing violence) (Table 5). This points to possible increases in the actions taken by community members in responding to VAW, including separating a couple that were fighting (increase in the mean from 57.2% to 63.2%; a total increase of 6%; *P* = 0.180), and reporting instances of VAW to relevant authorities (59.2 to 63.9 representing a mean increase of 4.7%; *P* = 0.278).

**Table 5:** Bystander interventions reported by participants at baseline and endline.

Participant agrees with the statement: ‘in the past 6 months, have you seen any village members doing any of the following actions…’	Baseline *n* (%)	Endline *n* (%)	*P*-value
Gathering people in your village to help when violence is occurring.	182 (65.7%)	154 (69.4%)	.386
Turning up at a home to distract or stop a couple from fighting.	153 (56.3%)	134 (60.6%)	.326
Separating a couple that were fighting.	158 (57.2%)	139 (63.2%)	.180
Reporting instances of violence against women to relevant authorities/leaders	164 (59.2%)	142 (63.9%)	.278
Participating in a village or church activity about violence against women.	179 (65.8%)	144 (65.2%)	.880
Helping a woman who is experiencing violence (e.g. by talking to/advising her or helping her to attend services).	208 (75.1%)	168 (76.4%)	.742
Confronting a man who is using violence.	92 (33.9%)	76 (34.7%)	.861
Speaking out against violence in the village.	79 (28.8%)	54 (24.4%)	.273
Hanging up or passing out materials like posters or flyers related to violence against women or happy relationships.	143 (52.0%)	117 (53.4)	.753
Implementing or enforcing village by-laws relating to violence against women.	198 (72.2%)	160 (74.1%)	.654

### The relevance of actions taken to address VAW

The actions taken by community members to respond to VAW were, by construction, relevant for the communities involved. All actions were undertaken on a voluntary basis, unpaid, and at the discretion of community members. Many community members made changes in their community during the pilot, including increasing support for local fruit sellers, increasing church attendance, and listening to youth (especially girls) during community forums. In one example, interviews with community members talked about the proactive involvement of *matai* in cases of violence:

The matai who were at the training helped to strengthen the wellbeing of everyone in the village like encouraging young men to have a plantation of their own to support and feed their families. Matai also went around visiting families during the day to make sure children are at school. There are hardly any drunken people during the night because once they get caught, they will be punished (72-year-old woman, village 07).

As described, the actions *matai* took in response to the pilot may not be directly related to reducing VAW. However, many of the actions taken by community members were linked either directly or indirectly to a theory of change for VAW prevention created by CBRs with their communities during the first 3 years of the EVE project. For instance, this theory of change explicitly linked keeping children in school with reduced VAW because of the role education plays as a protective factor for VAW perpetration and experience (Yakubovich et al. 2018).

Another example of actions linked to the theory of change were evening prayers, which play an important cultural role in Samoan villages. It is a time when *aiga* (family) can discuss family problems without the cultural prescriptions of family hierarchies and use prayer as a means of resolving conflict (including violence). Young men are often responsible for ensuring that community members adhere to curfews that mark the beginning of prayer time. In one community, all the young men in the village were responsible for this task and therefore unable to participate in evening prayers themselves. At the same time, the village was struggling with youth violence during evening prayers. Through their involvement in the pilot, the community decided to change their village bylaws to ensure that only three young men would patrol the village on any given day and all others would be at home with their *aiga*. This had an immediate impact on youth violence in the village, according to one young woman:

In our village, even those who previously didn't attend church are now actively participating, fostering love and unity among us. The programme's impact has been so profound that our community is now free from the youth violence that once plagued us. Three men now patrol during evening devotions, ensuring safety…The village council convenes meetings promptly whenever there's a case of violence. (24-year-old woman, village 06)

We also assessed whether the pilot was relevant for the community as a whole or only for certain groups, by disaggregating data by gender and age. As shown in Table 6, women attended more sessions than men on average, with 58% attending all six sessions as opposed to 36% of men.

**Table 6:** Participants in the pilot disaggregated by gender.

Village #	Attended 100%	Attended 100%	Attended 4+	Attended 4+	Attended 2+	Attended 2+	Lost to follow-up %
	Men	Women	Men	Women	Men	Women	
01	24%	81%	76%	94%	100%	100%	17%
02	4%	43%	38%	62%	79%	100%	43%
03	25%	20%	50%	56%	100%	100%	19%
04	30%	33%	60%	71%	100%	100%	22%
05	33%	73%	80%	100%	100%	100%	35%
06	40%	65%	80%	94%	100%	100%	0%
07	56%	47%	94%	79%	100%	100%	26%
08	67%	73%	78%	93%	100%	100%	38%
09	40%	80%	80%	93%	100%	100%	0%
10	38%	59%	63%	88%	100%	94%	23%
	**36%**	**58%**	**70%**	**83%**	**98%**	**99%**	**21%**

Bolded values are averages across all 10 villages.

Attendance also varied considerably across villages. Maximum attendance was 81% of women attending 100% of sessions (village 01) and 67% of men (village 08); and minimum attendance was 20% of women attending 100% of sessions (village 03) and 4% of men (village 02).

Individuals across a wide age range participated in the pilot with the average age of 42 (range 18–69; Table 2). This reflects the average age of individuals living in rural communities in Samoa with a loss of those in their 20s to work overseas or in the urban area.

During a planning workshop, CBRs decided that the pilot should include representatives from different positions in the hierarchy of Samoan villages (i.e. matai, untitled men/women, and youth). Community members discussed the inclusion of youth as a central advantage of the intervention design. While we did not collect information about gender identity from participants, nor did we collect data about disability, these identities are now the focus of a follow-up phase of the EVE Project.

### Addressing power structures arising from coloniality

The aim of the pilot was to address coloniality by facilitating more effective community decision-making about VAW prevention by providing the resources and tools needed to construct an approach to VAW prevention aligned with *fa’a Samoa*. This was successful in encouraging villages to take ownership of VAW prevention, designing activities that they felt would work, and that were closely aligned with *fa’a Samoa*.

Village bylaws were one example of this, as a Samoan mechanism for conflict resolution in village communities. Prior to the pilot, some villages had already created bylaws to punish members of the community who perpetrated violence. Village members discussed how these bylaws had been strengthened because of the pilot, which had offered new opportunities for the village to reevaluate what they had done previously and establish new protections for survivors:

The programme has allowed the revision of our village by-laws to punish the person who commits illegal acts. The village leadership is well ordained and has been honoured in these times. It has made people open up, especially those seeking support when they are abused. (35-year-old woman, village 09)

However, the effectiveness of village bylaws in reducing VAW is controversial in Samoa and requires more research to disentangle effectiveness. Some CBRs acknowledged in group discussions how village bylaws may also have negative impacts on VAW, by putting additional stress on families to pay fines and thereby increasing rather than decreasing violence. This was an ongoing debate during CBR workshops as others saw village bylaws as an effective deterrent to violent behaviours (see Samoa Office of the Ombudsman 2018, Lowe et al. 2023 for a detailed discussion).

Moreover, the pilot was less successful in addressing coloniality as a root cause of VAW (Segato and Monque 2021). As the pilot progressed, it became clear that Samoan communities have unique understandings of both ‘gender’ and ‘violence’ that arise from an Indigenous worldview, which were difficult to integrate into the pilot. There wasn’t an evaluated LMIC intervention to prevent VAW that had used a decoloniality framework for us to draw on. In brief, in Samoa, gender is recognized as a fluid concept with men’s and women’s roles interchanging over the life course (European Commission et al. 2016), and CBRs, as well as community members, contested definitions of gender that talked about prespecified roles, claiming that in Samoa, roles change over one’s lifetime.

### Limitations

While the EVE Pilot contributes to the potential of co-designing VAW prevention interventions with high-prevalence communities, it has limitations. First, community participants in the pilot were selected by CBRs as community leaders, and this may have contributed to a bias towards positive results. Social desirability bias may also have been a concern in the postintervention survey. Secondly, the modest sample size limited our power to clearly identify change over time in outcomes. In particular, the low postsurvey numbers (128 men participated in the baseline, whereas 89 participated in the endline survey, in contrast to 161 women in the baseline and 140 at endline) limited our ability to draw conclusions. Behavioural changes, including VAW reductions, may take more than the 6-month duration of our pilot intervention, however our pilot does demonstrate progress towards this goal in short time frames. A key component of this has been the co-design process adopted prior to the intervention, which, while labour-intensive, allowed us to establish trusting relationships. However, these same relationships, if properly built, may also contribute to contamination of the results for an experimental study, particularly in a small population such as Samoa. While we see this as a clear risk with a definitive trial, a pragmatic approach where such limitations are considered may offer an alternative option.

## Discussion

The EVE pilot demonstrates the potential of co-designing VAW prevention interventions with communities as part of policy development, as well as some challenges. The intervention effectively brought the issue of VAW to the surface of conversations in villages, inspiring *matai* to instigate activities they thought would help address the issue (i.e. keeping children in schools, encouraging church attendance, and evening prayers). This confirms evidence from other settings that village members do take action in responding to VAW (Lowe et al. 2022). Seeing communities as ‘in-deficit’ or ‘failing’ to reduce VAW, undermines their capacity to come up with their own solutions and bring about change (Bryant et al. 2021). In contrast, interventions that encourage communities to seek their own solutions to problems such as violence can motivate social change simply by asking the questions: ‘how could you do this better?’

Essentially, we suggest that researchers designing interventions from an outsider perspective may need to challenge their own assumptions about what strategies are most useful in reducing VAW, subverting the co-production literature that cites unpredictable outcomes as a disadvantage (Filipe et al. 2017, Ní Shé and Harrison 2021). The EVE Project highlights how a focus on unpredictable outcomes as the problem may be counterproductive to addressing the power dynamics involved in co-designing interventions, and sees this lack of predictability as a fundamental part of the process rather than something to correct (Iedema et al. 2010). We recognize that this can lead to discomfort on the part of the external ‘expert’ and a sense of ‘not knowing what is going on.’ However, it is precisely this discomfort that opens up new spaces for researchers to develop new positions (Chadwick 2021). As suggested by others, allowing new spaces for communities to find their own solutions to well established problems may be its own form of epistemic justice (Fricker 2007).

At the same time, we are cautious that this approach can lead to a form of cultural relativity and can also be used to undermine VAW prevention (i.e. by arguing that the acceptance of VAW is normal in certain cultural contexts). The EVE pilot equally highlights the need for evidence-based scaffolding to support communities in finding effective solutions. Without access to the evidence, communities will rely on what they have always done to prevent VAW, which rarely works in the ways they anticipate given the extent of the problem (Lowe et al. 2022, 2023). However, over the past 20 years, the field of VAW prevention has established growing evidence that supports the need for survivor-centred responses that directly challenge gendered social norms and the acceptance of violence at a community level (Jewkes 2014, Semahegn et al. 2019). The EVE Project shows the potential for sharing this evidence with communities—a form of south-to-south capacity strengthening—as part of a structured process of experiential learning and reflection. While the importance of experiential learning to effective VAW responses has recently been shown for frontline health workers (Allison et al. 2024), the EVE Project extends this evidence to CBRs responding to VAW in their own communities.

Overall, the EVE pilot was successful in co-creating solutions to the problem of violence that were relevant to community needs and contributed to community ownership of the problem. The development of a theory of change in partnership with participating communities played a key role in ensuring this (Mannell et al 2023a). However, while this was theorized as leading to reductions in VAW, the pilot revealed a mixed picture. Some of the gendered social norms held by women changed, while their experiences of violence did not. This was expected given the difficulties of changing the violent behaviours of men who were not involved in the project. However, the absence of a significant change on norms of violence by men points to either the small sample size (unable to detect the change) or a disconnect between norms of violence and related behaviours by men (Salmivalli and Voeten 2004). In contrast, the pilot’s borderline effectiveness in changing men’s physical perpetration of VAW provides hope for future interventions and highlights the need for interventions specifically focused on men’s behaviour as part of community programmes in Samoa.

The EVE pilot was less successful in decolonizing VAW prevention, raising questions as to whether this should even be an aim of co-design. Global health scholars, including ourselves, have long upheld the decolonizing potential of co-design (Turnhout et al. 2020, Khan et al. 2022). However, this assumes that the outcome of a co-production project is defined by communities for their own benefit, as suggested by Smith (1999), which often runs counter to addressing VAW in high-prevalence communities. In Samoa, VAW prevention is rarely a community-defined outcome; long-term work is needed for community members to see the ways in which VAW is harming their way of life and the options that exist for addressing it. This requires promoting a particular worldview—one where women are never deserving of violence and where the individual survivor should always be supported over community needs (Bunch 1990, Evatt 2002). The EVE pilot tried to balance a decolonial perspective with this anti-VAW perspective by introducing evidence-based interventions that had been effective in other countries in the Global South. However, such interventions draw on similar discourses of individual human rights without recognition of the ways in which they come into conflict with communitarian rights and responsibilities, particularly in the Pacific (European Commission et al. 2016).

A decolonial perspective directly challenges such universal or normalizing discourses, and points to why co-production may be insufficient as a form of decolonization for VAW prevention interventions in Samoa. Mignolo and Walsh (2018) define decolonization, or *decoloniality*, as a process of epistemic delinking from the colonial matrix of power that normalizes particular discourses to the exclusion of others. To do this, they call for *pluriversality* as opposed to universality, which allows for the coexistence of different ways of thinking. Accomplishing this is a far more profound process than challenging community norms of VAW as a part of intervention co-development, and it also goes beyond defining outcomes that align with community needs. To decolonize VAW, we first need a critique of how the colonial matrix of power has contributed to dominant discourses of violence in women’s lives, followed by an alternative discourse informed by an Indigenous worldview.

## Conclusions

Co-designing interventions to reduce VAW is possible and may offer a viable policy strategy for reducing VAW in high-prevalence settings. However, it also requires considerable flexibility in allowing communities to define the context-specific mechanisms through which change occurs. This is both an opportunity and a potential risk. The EVE pilot demonstrates how taking an insider or Indigenous standpoint is integral to creating the potential for new mechanisms to emerge from the co-design process, including local ownership and long-term sustainability.

## Data Availability

The data underlying this article will be shared on reasonable request to the corresponding author.

## Reflexivity statement

The authors include six women and two men, spanning different levels of seniority, qualifications, and privilege. Five authors are geographically based in Samoa where the study took place, while three authors are based in the UK and are affiliated with UCL as a research partner. One author is a leading statistician in global health, while the seven remaining authors are experts in the prevention of violence against women from both an academic and practitioner standpoint.
